# Diffusion Dynamics of Volatile Organic Compounds into Integrated Surface-Supported Metal–Organic Frameworks Heterojunctions

**DOI:** 10.1021/acsami.5c16835

**Published:** 2025-12-22

**Authors:** Thamiris Cescon dos Santos, Wagner Wlysses Rodrigues de Araujo, Tatiana Parra Vello, Carlos Vinícius Santos Batista, Luiz Gustavo Simão Albano, Carlos César Bof Bufon

**Affiliations:** † Brazilian Nanotechnology National Laboratory (LNNano), Brazilian Center for Research in Energy and Materials (CNPEM), Campinas, São Paulo 13083-970, Brazil; ‡ Mackenzie Institute for Research in Graphene and Nanotechnologies (MackGraphe), Mackenzie Presbyterian Institute (IPM), São Paulo 01302-907, Brazil; § Postgraduate Program in Materials Science and Technology (POSMAT), 736790 São Paulo State University (UNESP), Bauru, São Paulo 17033-360, Brazil; ∥ Department of Physics, Institute of Geosciences and Exact Sciences, São Paulo State University (UNESP), Rio Claro, São Paulo 13506-900, Brazil

**Keywords:** HKUST-1, SURMOF, heterojunctions, volatile organic compound, molecular sensing

## Abstract

Surface-supported metal–organic frameworks (SURMOFs) have emerged as promising hybrid materials across diverse applications, including gas separation, energy storage, catalysis, and sensing. These capabilities are primarily associated with their high porosity and reasonable control over crystallinity. However, the diffusion of volatile organic compounds (VOCs) in these structures remains poorly understood, limiting their range in strategic applications. One significant challenge is developing effective integration approaches that enable precise control of molecular transport in these structures. In this work, we investigated the diffusion dynamics of various VOCs (methanol, ethanol, propanol, and hexane) into two-terminal devices based on a HKUST-1 thin-film SURMOF, using conventional photolithography and nanomembrane-origami technology. Systematic electrical responses (DC and AC) were monitored as precise and tunable tools for VOC differentiation. Our experimental results align with Gao’s model, offering new insights into the diffusivity, permeability, pore accessibility, and intracrystalline diffusion lengths of VOCs within integrated HKUST-1 with thicknesses below 70 nm. These findings represent significant advances in understanding diffusion processes in monolithically integrated nanoporous materials, with potential implications for future innovations in molecular sensing and environmental monitoring technologies.

## Graphical abstract

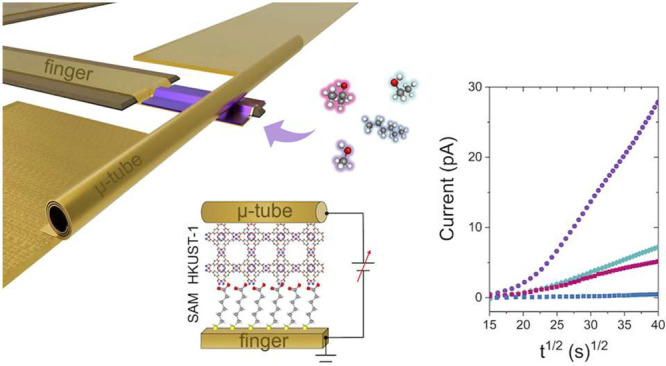

## Introduction

Metal–Organic Frameworks (MOFs) are hybrid materials widely recognized for their crystallinity, porous structure, and high surface area.^1^ The assembly of inorganic clusters with organic linker molecules allows a significant range of structures and compositional modulation, turning these materials highly versatile.^2,3^ Monolithic thin-films, known as Surface-Supported Metal–Organic Frameworks (SURMOFs), are synthesized by epitaxial liquid-phase deposition and retain the intrinsic properties of MOFs while offering the advantage of integration into nanoscale devices with tailored crystallinity.^4,5^ These characteristics significantly expand the technological potential of these materials.

Porous materials have been extensively explored for mass separation,^6^ catalytic conversion,^7^ selective adsorption,^8^ and sensing.^9,10^ Advances in these fields have strongly driven research into the interactions between guest molecules and pore walls,^11,12^ as molecular transport directly impacts performance.^13,14^ In this regard, MOFs and SURMOFs provide a periodic and crystalline nanoenvironment to host guest molecules, enabling precise spectroscopic and physicochemical studies.^15,16^ Moreover, the monolithic nature of SURMOFs provides a substantial advantage over other porous materials, which often exhibit structural inhomogeneities that hinder an engineered environment for hosting guest molecules.^17^

HKUST-1 is a metal–organic framework comprising copper ions and benzene-1,3,5-tricarboxylic acid arranged in a paddlewheel structure.^18^ It is one of the most extensively studied MOF structures,^19–22^ making it suitable for various practical applications. The ligand arrangement results in two coordinatively unsaturated Cu sites per paddlewheel, allowing them to interact with polar molecules.^23^ Furthermore, HKUST-1 exhibits a remarkable surface area and pronounced porosity, making it a promising platform for molecular sensing applications.^24,25^

Despite their potential, theoretical insights describing transport phenomena in such materials remain open.^26–28^ Intracrystalline diffusion has been considered the primary mechanism governing mass transfer in nanoporous materials.^29,30^ Additionally, imaging techniques such as interference microscopy and infrared microscopy have demonstrated the presence of surface barriers in specific nanoporous materials.^31,32^ The origin of surface barriers is not yet fully understood^33^ and may be partially attributed to pore obstruction, misalignment, surface guest–host interactions, or a combination of these effects.^34,35^

Although imaging techniques have provided valuable insights, they usually do not enable device-compatible diffusion analysis. In this context, electrical characterization methods enable the investigation of diffusion dynamics in such structures. Most analyses predominantly rely on the quartz crystal microbalance (QCM), a highly complex technique.^36–38^ This limitation — particularly relevant when using MOFs as active sensing layers — stems primarily from the challenges of integrating them into device architectures, as their synthesis is often incompatible with conventional fabrication processes.^39^ Moreover, electrical methods offer key advantages over imaging methods, including real-time monitoring and high sensitivity to minor variations in analyte concentration. Understanding the interplay between electrical response and molecular diffusion in MOF thin-films is crucial for developing high-performance MOF-based sensors and electronic devices.^40,41^ Recently, we addressed these challenges by integrating SURMOFs into a robust electronic platform,^42^ which enabled their detailed electrical characterization. This approach provides unique insights that complement — and, in some cases, surpass — those from traditional spectroscopic and imaging techniques.

Gao et al.^43^ proposed a theoretical model for the uptake rate based on surface permeability,^32^ providing a refined understanding of surface barriers and intracrystalline diffusion. This approach offers a distinct advantage over existing models by eliminating the need for prior knowledge of intracrystalline diffusion coefficients or constraints related to crystal size and morphology. As a result, it enables independent analysis of surface barriers and intracrystalline diffusion. Applying Gao’s model,^43^ we systematically study the diffusion of volatile organic compounds (VOCs) in HKUST-1-based devices, providing quantitative insights into diffusivity, permeability, pore accessibility, and intracrystalline diffusion lengths.

To achieve this, the HKUST-1 thin-films were integrated into a two-terminal vertical junction structure using standard microfabrication techniques. The fabrication employed well-established nanomembrane-origami technology,^44–46^ which ensures self-adjustable electrical contacts, high reproducibility, and preservation of the active layer’s structural integrity. Device characterization was performed through standard electrical measurements, including current–voltage (*I*–*V*) characteristics, transient current (*I*–*t*) responses, and capacitance-frequency (*C*–*f*) measurements under varying VOC concentrations.

Using Gao’s model, we analyzed the diffusion and surface-barrier processes for methanol, ethanol, propanol, and hexane in the HKUST-1 thin-film. The influence of molecular properties, including polarity, dipole moment, kinetic diameter, and molecular weight, on diffusion behavior was systematically evaluated and compared with theoretical predictions. Furthermore, we demonstrate that electrical current gain and capacitance variations are effective discriminators for different analytes.

Our findings represent a significant advancement in understanding diffusion processes within monolithically integrated nanoporous materials. This work not only delivers key insights for optimizing SURMOF-based sensors but also lays the groundwork for the development of next-generation functional technologies leveraging engineered nanopores.

## Experimental Section

### Chemicals

Chemicals were purchased from commercial suppliers and used without further refinement. Copper acetate (II) (CuAc, 98%), trimesic acid (BTC, 95%), and 16-mercaptohexadecanoic acid (MHDA, 90%) were obtained from Sigma-Aldrich, São Paulo, Brazil. Glacial acetic acid (99.7%) and acetone from Synth, São Paulo, Brazil. Ethanol (99.5%), hexane (95%), isopropyl alcohol (99.8%), and methyl alcohol (99.9%) were obtained from Merck Millipore, Darmstadt, Germany.

### Device Fabrication

Vertical heterojunctions were fabricated following the approach described in our previous contributions.^42,44,45^ The fabrication steps were based on conventional photolithography (using AZ 5214E photoresist), illustrated in detail in Figure S1. Over a 2 μm-thick SiO_2_ on a Si wafer, a “mesa” structure was patterned by removing 190 nm of SiO_2_ by reactive ion etching (using CF_4_ gas as a reactant), Figure S1a. Then, Cr/Au (5/10 nm) metallic layers were deposited at 0.5 Å/s to create the bottom electrode (finger-like electrode), as shown in Figure S1b. Next, a Ge layer (20 nm) was deposited at a rate of 0.2–0.3 Å/s (Figure S1c) and then subjected to a chamber with high humidity (∼90%) for 72 h to oxidize the Ge into GeO_*x*_, creating an aqueous-soluble sacrificial layer. Afterward, the strained nanomembrane containing Au/Ti/Cr (5/15/20 nm, deposited at 0.5/1/5–6 Å/s, respectively) was patterned on top of the GeO_*x*_ sacrificial layer (Figure S1d). Finally, the contact pads, Cr/Au (20/50 nm at 1 Å/s), were patterned, as shown in Figure S1e. All metallic layers were deposited using electron-beam evaporation. In the final stages of device fabrication, sequential etching and rolling processes form vertical heterojunctions, as illustrated in Figure S1f–h.

### SAM Immobilization and HKUST-1 Growth

The as-fabricated devices were functionalized with self-assembled monolayers (SAMs) by immersing them in a 10% (v/v) acetic acid-ethanol solution containing 0.5 mM MHDA for 20 h. The solution was maintained at 50 °C for 1 h, then allowed to react for an additional 19 h at room temperature. The devices were washed with ethanol at 50 °C to remove nonimmobilized molecules, then dried under an N_2_ stream. HKUST-1 growth was achieved through a layer-by-layer (LbL) quasi-liquid-phase epitaxial process.^42,44,45^ This involved alternating immersion cycles of functionalized devices in ethanolic solutions of 1 mM CuAc (2 min) and 1 mM BTC (4 min), with intermediate ethanol rinses (1 min prewash and 5 min main wash) between each precursor immersion. The procedure was repeated for 25 cycles. Then, the HKUST-1 devices were washed in ethanol, dried under N_2_, and stored in a desiccator for at least 24 h to remove the trapped solvent from the pores. The detailed growth process is illustrated in Figure S2.

### HKUST-1 Patterning and Rolled-Up Process

After HKUST-1 growth, the devices’ active area was protected by a photoresist (Figure S3a). Then, SURMOF from unprotected regions was removed using O_2_ plasma surface treatment (90 W, 3 mbar) for 10 min, as shown in Figure S3b. A trench was patterned in the nanomembrane region to control the rolled-up process precisely (Figure S3c). In the sequence, the GeO_*x*_ sacrificial layer was dissolved in aqueous solution (0.5% (v/v) H_2_O_2_). The dissolution of the sacrificial layer releases the strained nanomembrane, resulting in a μ-tube that is rolled-up until it reaches the trench limit (Figure S3d). Then, the photoresist was removed in acetone, allowing the μ-tube to reach the top of the SURMOF HKUST-1 thin-film (Figure S3e). Afterward, the devices were immersed in water/ethanol (20/80% v/v) solution to complete the rolled-up process. The integrity of HKUST-1 after such processes was previously verified in detail.^47^ Finally, the devices were dried on a hot plate at 100 °C and stored in a desiccator for 3 days before the electrical characterization. Figure S3f shows a dark-field microscope image in which the SURMOF growth and the etched region can be verified.

### VOC Loading and Electrical Characterization

The electrical characterization was conducted using a Semiconductor Parameter Analyzer 4200-SCS from Keithley (USA), which has a minimum current threshold of 10^–14^ A. Current–voltage (*I*–*V*) and current–time (*I*–*t*) curves were obtained using a conventional probe station equipped with micromanipulators and tungsten tips. A custom-built chamber with controlled N_2_ flow was integrated into the probe station system to achieve the same initial inert atmosphere with humidity below 4%. Methanol, ethanol, propanol, and hexane atmospheres were achieved using four reservoirs containing 5 mL of each compound, as shown in Figure S4. All VOC vapors were generated from anhydrous solvents (≥99.8%) and maintained a relative humidity below 5%. These precautions ensured that the measured responses originated solely from VOC adsorption and diffusion, with negligible interference from water vapor. The *I*–*V* cycles were performed from −2 to 2 V, with a 10 mV increment. An MFIA Impedance Analyzer from Zurich Instruments acquired capacitance-frequency (*C*–*f*) curves. A sine-wave voltage of 100 mV amplitude (with no DC offset) was applied, and data were acquired at 10 points per decade over a frequency range from 1 MHz to 100 mHz.

### Complementary Characterization

X-ray Diffraction (XRD) was carried out using a diffractometer D8 Advance Focus Bruker AXS equipped with primary and secondary Soller slits of 2.5° divergence and antiscattering. Diffractograms of HKUST-1 were acquired from 4 to 16° (2θ), with a step size of 0.015°, time per step of 50.0 s/°, and incident angle of 0.20°, using a Cu anode (λ = 0.154 nm) operated at 40 kV and 20 mA. The diffractogram from the Au substrate was acquired as a reference, with θ/2θ ranging from 68° to 70.5°, a step size of 0.015°, and a time per step of 0.1 s/°. Raman spectra were acquired using a WITec Raman microscope equipped with a 600 g/mm grating, a 532 nm excitation laser, and a 100× objective. Each spectrum consisted of ten 30 s accumulations at a laser power of 1 mW. The Au/SAM-HKUST-1 spectra were acquired before and after a 16 h VOC exposure. The sample was removed from the sealed container and immediately subjected to Raman measurements. The results presented refer to the same sample region after each VOC exposure. Atomic Force Microscopy (AFM) images were obtained using a Dimension Icon Instrument (Bruker) in peak force tapping mode with a Si cantilever (190 kHz resonance) and a Pt/Ir-coated tip (25 nm radius). The surface potential was measured using Kelvin Probe Force Microscopy (KPFM) in dual-pass mode. A 0.5 V AC voltage was applied at 500 Hz, maintaining a lift height of 50 nm. Both AFM and KPFM data sets were subsequently processed using Gwyddion software. Due to the sub-100 nm thickness and the integration onto conductive substrates, nitrogen adsorption/desorption analyses were not applicable. Instead, AFM and KPFM provided nanoscale insights into the film morphology and electronic uniformity, serving as effective tools for assessing the continuity and homogeneity of the HKUST-1 SURMOF (Figure S5a–c). Thicker HKUST-1 films (∼200 nm, 85 LbL cycles) were employed for XRD and Raman measurements to enhance signal intensity. In comparison, functional devices and diffusion studies were conducted on thinner films (∼70 nm, 25 LbL cycles) that share the same crystallographic orientation and growth direction.^42,44,45^

### Differential Evolution Algorithm

Differential Evolution (DE) is a stochastic, population-based optimization algorithm designed for solving complex global optimization problems. In DE, each candidate solution is represented as a vector of real-valued parameters corresponding to the variables in the objective function. The algorithm iteratively evolves a population of such vectors through three key operations: mutation, crossover, and selection. These operations collectively enable efficient exploration of the solution space, driving the population toward the global optimum.^48–51^

In each generation, new candidate solutions (called trial vectors) are generated by mutating existing solutions. A widely used strategy, known as best/1/bin,^50^ operates by selecting the current best-performing individual (*b*_0_) and adding a weighted difference between two randomly selected population members (*b*_1_ and *b*_2_). The mutation step follows:

$$b^{\prime }=b_{0}+F\times (b_{1}-b_{2})$$ 1

where *F* is the mutation scaling factor (typically between 0.5 and 1), controlling the amplification of the differential variation. This step promotes diversity and helps the algorithm explore the parameter space effectively. Next, the crossover step mixes the mutated trial vector (*b*′) with the original candidate vector. Each parameter in the trial vector is either inherited from *b*′ or retained from the original candidate, based on a probability defined by the crossover rate (CR, typically between 0 and 1). Specifically, for each parameter, a random number between 0 and 1 is drawn; if this number is less than CR, the parameter is taken from *b*′; otherwise, it is taken from the original. At least one parameter is always inherited from *b*′, ensuring that the trial vector differs from the original. The selection step then compares the trial vector with the original candidate. The one with the better objective function value (i.e., lower error or higher fitness) proceeds to the next generation. If the trial vector also outperforms the best solution found so far, it replaces it as the new global best.^48–51^ In this work, DE was employed to optimize the parameters of eq 4, which models transient current responses as a function of the square root of time. The objective was to minimize the difference between the experimental current data and the theoretical model, enabling accurate extraction of key electrochemical transport parameters: the intracrystalline diffusivity (*D*), the surface permeability (α), and the surface barrier (α/*l*).

## Results and Discussion

Figure 1 depicts our strategy for studying the diffusion dynamics of selected VOC compounds on LbL HKUST-1 thin-films integrated into a rolled-up nanomembrane device architecture. The details regarding LbL growth and device fabrication steps can be found in the experimental section, Supporting Information (SI, Figures S1–S3), and our previous contributions.^42,44,45^ After the sequential device fabrication steps (Figure S1), the LbL growth method is performed, as shown in Figures 1a and S2. The functionalized gold surface ensures that the first layers are efficiently anchored.^4^ In this case, the carboxylic tail group of the SAM binds to the CuAc clusters, which coordinate with BTC molecules.^47^ Then, by intercalating CuAc and BTC precursors, the thickness of HKUST-1 can be precisely adjusted. The process was repeated for 25 growth cycles. Figure 1b illustrates a schematic representation of the device concept, which enables a robust, self-adjusting electrical top contact via strain engineering of selected metallic thin-films and directional sacrificial layer removal. The architecture is highly versatile for various roles,^52–54^ particularly in ultrathin, highly porous materials.^42,44,45^ The typical HKUST-1 pore sizes of 14 and 10 Å allow the penetration and diffusion of various small molecules.^23^ As illustrated in Figure 1c, the HKUST-1 was systematically exposed to four different VOC compounds: methanol, ethanol, propanol, and hexane.

**Figure 1:**
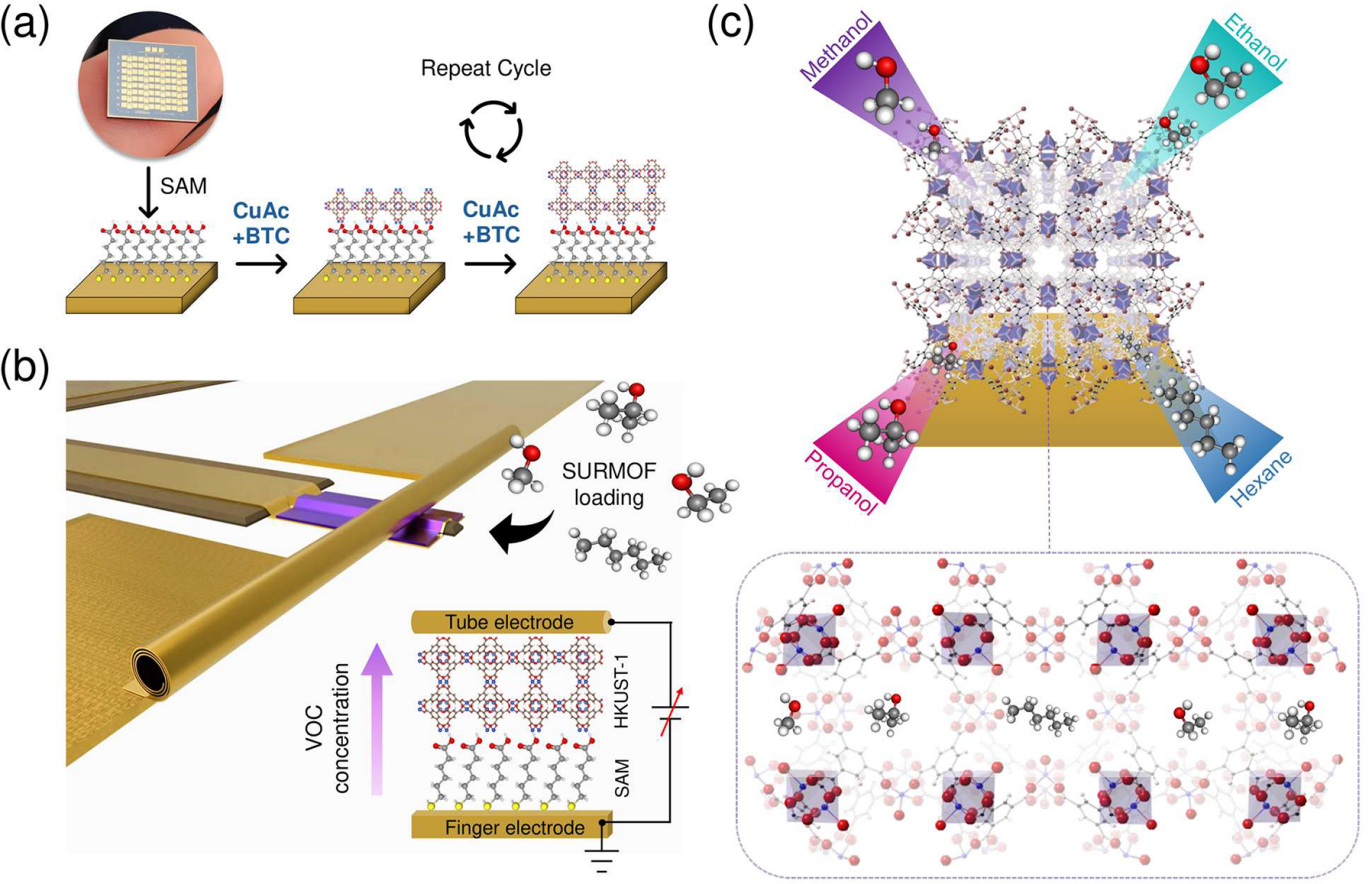
Schematic illustration of HKUST-1 growth and device integration. (a) Substrate functionalization and layer-by-layer (LbL) growth. (b) Illustration of a rolled-up nanomembrane device, including the device cross-section and electrical circuit diagram (inset). (c) Representation of methanol, ethanol, propanol, and hexane molecules loading HKUST-1 pores.

Figure 2 exhibits the structural and chemical characterization of the HKUST-1 thin-film. An optical microscope image of a single Au/HKUST-1/Au rolled-up vertical heterojunction is shown in Figure 2a. The diffraction pattern presented in Figure 2b confirms the crystallinity of HKUST-1 after the optimized LbL growth process. The presence of two main peaks at 6.91° and 11.77° (2θ degree) reveals the growth orientations corresponding to planes (200) and (222), associated with growth directions [100] and [111] respectively, in agreement with previously reported results.^42,44,45^ The AFM image in Figure 2c (top) was obtained from the device’s active area (dotted rectangle) indicated in Figure 2a. The SURMOF thin-film exhibits a relatively homogeneous surface with a root-mean-square (RMS) of 22.4 ± 0.3 nm — a value significantly higher than those reported in previous studies, likely due to the increased number of LbL growth cycles.^42,44,45^ Thickness analysis, derived from the dotted profile line (Figure 2c, top), yields an estimate of approximately 70 nm (Figure 2c, bottom). Additionally, the KPFM analyses were performed on the device’s active area to map the surface potential of the HKUST-1 in detail. The results align with prior reports,^42^ corroborating the material’s electronic homogeneity and uniform surface potential distribution (Figure S5a–c).

**Figure 2:**
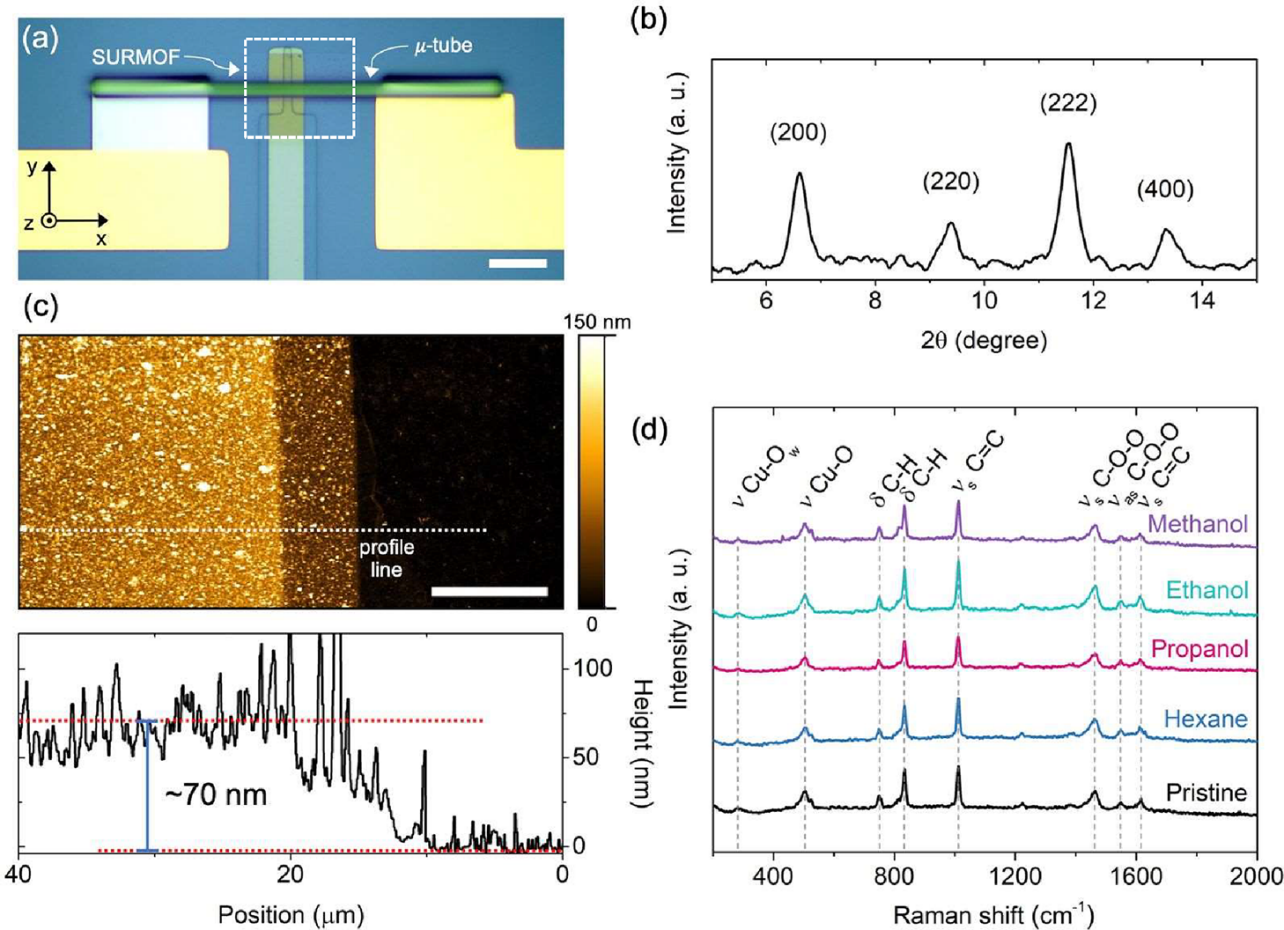
Structural and chemical characterizations of HKUST-1 thin-film. (a) Optical microscope image of Au/HKUST-1/Au rolled-up vertical heterojunction. (b) XRD pattern of a 200 nm thick HKUST-1 film (85 LbL cycles). (c) AFM topography image (top) of Au/HKUST-1 region; the profile height (bottom) is represented by the white dotted line. (d) Raman spectra of a 200 nm thick HKUST-1 film before (pristine) and after VOC (methanol, ethanol, propanol, and hexane) loadings. The scale bar in (a) is 40 and (c) 10 μm.

The stability of HKUST-1 under various VOC environments was analyzed using Raman spectroscopy (Figure 2d), with data collected after 16 h of exposure. The spectrum shows characteristic peaks at 276 cm^–1^ (νCu–O_w_, stretching), where O_w_ is the oxygen adsorbed on Cu^2+^,^55^ and a double band at 449–502 cm^–1^(νCu–O carboxylate stretching), along with benzene ring vibrations between 700 and 1100 cm^–1^(δC–H out-of-plane deformation mode at 745/828 cm^–1^ and ν_s_ C=C symmetric stretching mode at 1006 cm^–1^). Additional spectral features include the symmetric C–O–O stretching mode (ν_s_, 1460 cm^–1^), asymmetric C–O–O stretching mode (ν_as_, 1544 cm^–1^), and the benzene ring’s symmetric C=C stretching mode (ν_s_, 1616 cm^–1^).^55,56^ Crucially, the postexposure spectra retain the pristine HKUST-1 profile after N_2_ purging, confirming structural stability and demonstrating the material’s suitability for VOC sensing applications.

The AFM topography confirms the uniform and continuous nature of the SURMOF thin-film, while KPFM maps (Figure S5) reveal a homogeneous surface potential distribution, indicating consistent coverage across the active area. These observations, together with the XRD and Raman data, validate the film’s crystallinity, morphological uniformity, and structural stability, complementing the expected microporous characteristics of HKUST-1.

The electrical properties of Au/HKUST-1/Au rolled-up vertical heterojunctions were characterized utilizing a custom-built chamber integrated with a standard probe station (Figure S4a). The chamber was equipped with N_2_ inlets and outlets for precise humidity control, which was monitored in real-time using a commercial sensor (Figure S4b). A saturated VOC atmosphere was maintained using a liquid reservoir with a calibrated volume (see Experimental Section). Before measurements, preliminary *I*–*V* sweeps were conducted at varying humidity levels to verify the stability of the environmental control.

Under controlled N_2_ and humidity conditions, the devices exhibited a reversible increase in current of up to 2 orders of magnitude (Figure S6a). A pronounced hysteresis was observed during stepwise RH modulation up to 75% (Figure S6b), indicating humidity-dependent charge transport behavior. An additional hysteresis effect observed for pristine HKUST-1 devices is likely to originate from the interaction of structural defects (up to 2% Cu^+^ species) with humidity, which induces midgap states in the HKUST-1 band structure — thereby resulting in hysteresis under a reserved applied bias.^44,45^ Remarkably, the devices fully retained their baseline electrical characteristics after VOC exposure (Figure S7a), demonstrating exceptional stability. Also, these curves show evidence of negative differential resistance (NDR) around 0.25 V, likely associated with residual water molecules trapped inside the pores after data acquisition under humidity, which induce midgap states in the HKUST-1 band structure, which are accessible by an applied electric field.^45^ Moreover, the electrical responses were highly consistent across multiple devices on the same chip (Figure S7b), confirming excellent reproducibility.

Although ex-situ XRD after VOC exposure could further confirm long-term crystallinity, the Raman spectra (Figure 2d) already demonstrate the retention of all characteristic HKUST-1 vibrational modes, confirming the preservation of the Cu^2^^+^–BTC coordination network. In addition, the electrical measurements revealed complete recovery of the baseline current and hysteresis (Figures S6 and S7), indicating that both the structural and electronic properties remained unchanged after prolonged VOC exposure. Together with previous reports showing the chemical robustness of LbL-fabricated HKUST-1 SURMOFs,^42,44,45,47^ these results validate the framework’s structural stability under operational conditions.

Figure 3a–d presents the electrical responses of the devices under exposure to different VOCs. The N_2_ baseline curves correspond to a pristine state in which HKUST-1 pores remain unfilled. Hysteretic *I*–*V* behavior was observed until current saturation was achieved, with a magnitude of saturated current (@ 2 V) being VOC-dependent. Notably, the number of cycles required to reach saturation varied significantly. Hexane, for instance, achieved saturation with the fourth cycle, likely due to its higher volatility compared to the other tested VOCs. The distinct hysteresis patterns further suggest that VOC adsorption within the HKUST-1 pores modulates the devices’ capacitive properties, reflecting charge storage and transport dynamics. The possibility of competitive adsorption between water and VOC molecules was minimized by performing all tests under low-humidity conditions (<5% RH), using anhydrous solvents, and continuously purging the chamber with dry nitrogen before and after each VOC exposure cycle. The consistent and fully reversible electrical responses, together with the preserved Raman signatures after multiple exposure cycles, confirm that the diffusion behavior arises from VOC uptake rather than from water coadsorption or hydration effects.

**Figure 3:**
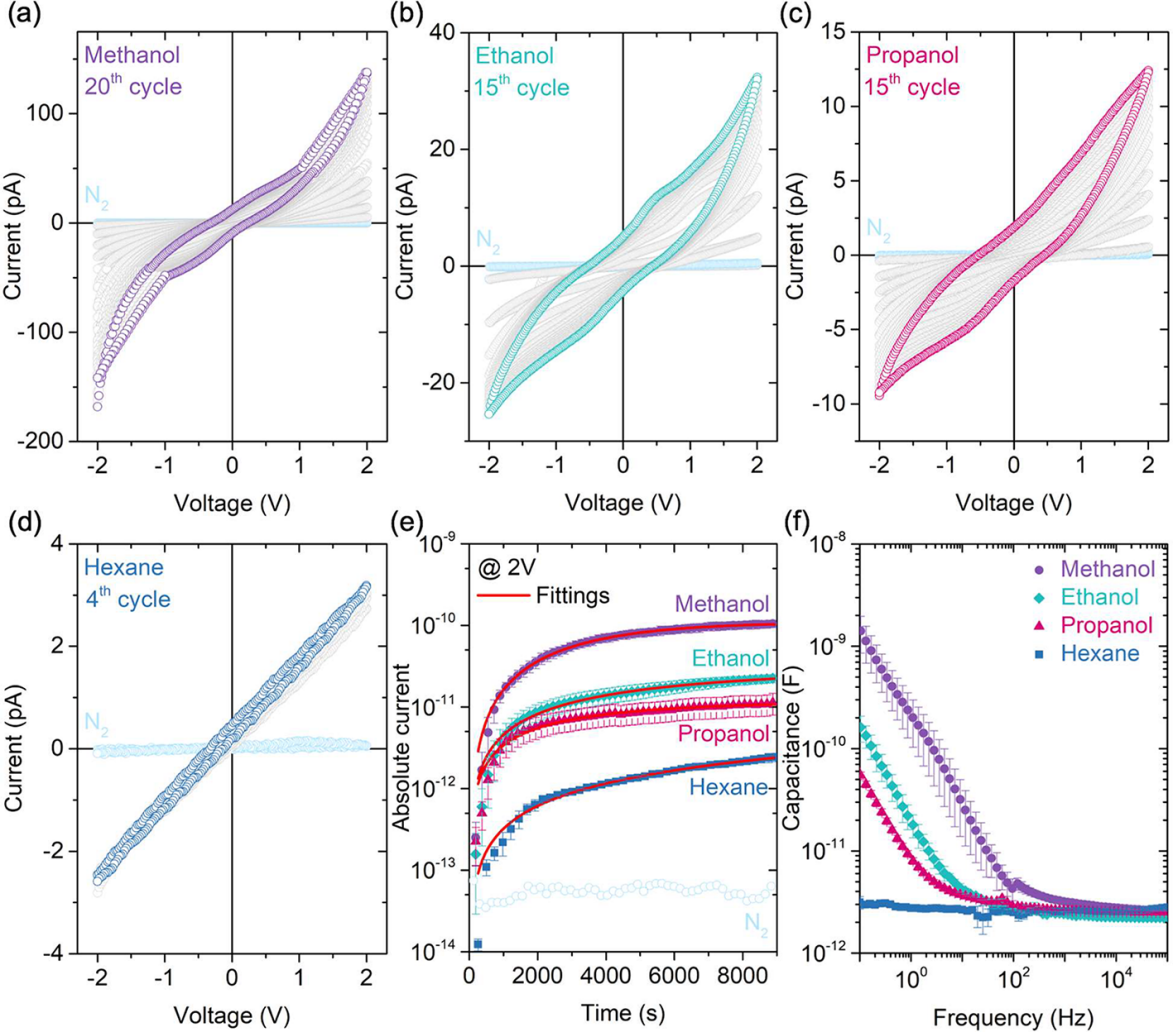
(a–d) *I*–*V* characteristics showing hysteretic behavior during cyclic measurements of (a) methanol, (b) ethanol, (c) propanol, and (d) hexane. (e) *I*–*t* measurements demonstrate the transient response until current saturation is achieved for each VOC. The experimental data were fitted using the stretched exponential Kohlrausch function (solid lines). (f) *C*–*f* characteristics of triplicate devices measured at VOC saturation.

Figure 3e displays the temporal evolution of current for each VOC under a constant 2 V bias. To gain an insight into the diffusive nature of the observed transient processes, we performed an analysis based on Graham’s Law (Figure S8).^57^ While Graham’s Law provides an empirical framework for gas diffusion through an aperture based solely on molecular mass,^58^ we employed this simplified approach exclusively to corroborate that the electrical responses reflect the VOC diffusion process. The diffusion parameters were extracted by plotting the natural logarithm of the transient current as a function of the square root of time, from which the linear region was fitted (Figure S8a). The resulting slopes and associated errors, summarized in Table S1, were then used to estimate relative diffusion rates and compared to the molecular weight dependence predicted by Graham’s Law (Figure S8b). It should be noted that this theory cannot fully capture diffusion in complex nanoporous systems, such as MOFs,^59^ where chemical interactions and spatial confinement become decisive factors.^60–62^

As evident in Figure 3e, the devices exhibit distinct saturation levels under different VOC atmospheres, with methanol achieving the highest saturation, followed by ethanol and propanol. While hexane exposure did not yield complete saturation, the experimental data showed reasonable agreement with the fitted curve. Error bars derived from triplicate measurements for each VOC confirm the statistical significance of these observations, as they remain nonoverlapping at saturation.

Notably, the initial responses to ethanol and propanol show overlapping error bars (*t* < 30 s), which we attribute to their similar molecular structures and comparable diffusion kinetics during the initial adsorption phase. The temporal current profiles deviate from conventional exponential behavior and are instead well described by a stretched exponential Kohlrausch function^63–65^ (eq 2), suggesting complex and dispersive transport mechanisms within the MOF framework.

$$I\left(t\right)=I_{0}\left(1-\exp\left[-\left({\frac{\left(t\right)}{\tau }}^{\beta }\right)\right]\right)$$ 2

where *t* represents the elapsed time during VOC diffusion into the HKUST-1 pores, *I*(*t*) denotes the time-dependent electrical current measurement, *I*_0_, corresponds to the equilibrium saturation current, τ is the characteristic loading time (relaxation time constant), and β is the stretching exponent, quantifying the dispersion of relaxation times in the system. The stretching exponent β (0 < β ≤ 1) in the Kohlrausch function quantifies the nature of diffusion processes, where β = 1 represents ideal Debye relaxation (homogeneous diffusion with a single characteristic time scale). At the same time, β < 1 indicates dispersive transport with distributed relaxation times, characteristic of heterogeneous diffusion in porous frameworks.^63,64^ This deviation from simple exponential behavior reflects the complex interplay of multiple adsorption sites and varied diffusion pathways within the MOF structure.

Eq 2 describes relaxation processes, representing the transition of a nonequilibrium system toward equilibrium.^63^ In our experiments, the relaxation dynamics are governed by VOC concentration gradients within the HKUST-1 pores, which we monitor through the electrical current saturation.

The curve fitting of the transient responses (Figure 3e) reveals characteristic times (τ) on the order of 10^3^ s for methanol, ethanol, and propanol, consistent with relatively rapid diffusion kinetics.^63^ In contrast, hexane exhibits a markedly longer characteristic time (∼10^7^ s). Despite hexane’s higher volatility, its slower kinetics suggest either (i) weaker host–guest interactions or (ii) more constrained diffusion pathways within the MOF structure.

The stretching exponent β characterizes the distribution of relaxation times within the system. Typically, values of β between 0 and 1 indicate a broad distribution of time scales, reflecting disordered or heterogeneous systems governed by diffusion-limited processes.^66^ In contrast, a β > 1 suggests a narrow distribution, implying a more uniform and rapid system response to external stimuli. For ethanol, propanol, and hexane, we obtained β = 0.97, 0.91, and 0.90, respectively, consistent with a moderately broad distribution of diffusion times. Although the stretching exponent β typically ranges between 0 and 1 for systems exhibiting dispersive diffusion, the value obtained for methanol (β = 1.49) suggests a more uniform and rapid saturation process. This deviation from conventional behavior may reflect the highly efficient, homogeneous pore filling enabled by methanol’s small size and strong interactions with the HKUST-1 framework.

Although conventional vapor adsorption–desorption measurements do not apply to the present thin-film devices because of the nanogram-scale mass of HKUST-1 (<100 nm thickness), the adsorption behavior can be inferred directly from the electrical responses. The transient current (*I*–*t*) profiles represent the time-dependent molecular uptake within the pores, while the capacitance–frequency (*C*–*f*) data capture the dielectric polarization associated with VOC adsorption and desorption. These measurements serve as in situ functional analogs of vapor adsorption tests, providing quantitative parameters of diffusivity (*D*) and surface permeability (α) through Gao’s model analysis.

The devices exhibited distinct capacitance responses at different frequencies, particularly below 10^2^ Hz (Figure 3f). This behavior is further elucidated in Figure 4a, where electrostatic potential maps and dipole moments reveal significant polarity variations among respective VOCs. Methanol, possessing the highest dipole moment, displayed the strongest capacitance response, while nonpolar hexane showed negligible activity. These low-frequency differences arise primarily from dipolar and interfacial polarization mechanisms, which dominate under slow alternating electric fields.^67^

**Figure 4:**
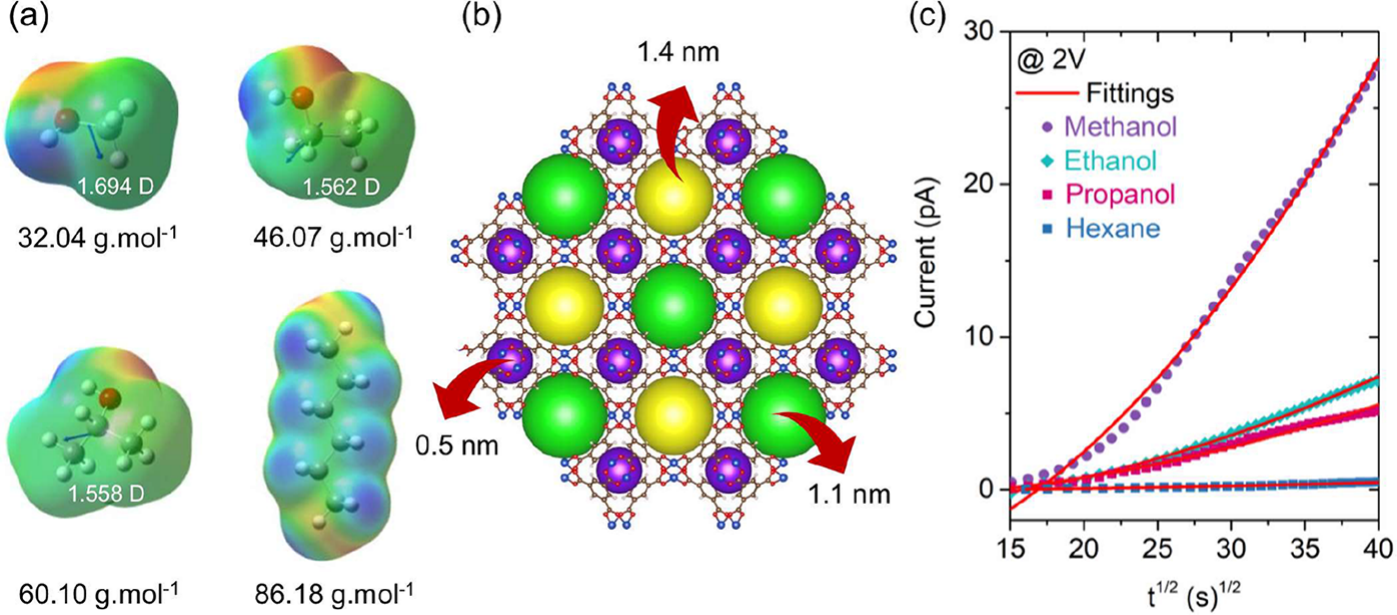
(a) Molecular properties of adsorbed VOCs: polarity, dipole moment, and molecular weight for methanol, ethanol, propanol, and hexane. (b) Pore size distribution in HKUST-1 (supercell representation). (c) Transient current response versus *t* ^1/2^ during VOC loading, with fitting curve from Gao’s diffusion model.

At frequencies above 10^2^ Hz, the capacitance values of all VOCs converge to a similar range. This behavior indicates that once the frequency exceeds the characteristic relaxation times of the polarization mechanisms, the influence of intrinsic molecular properties—such as dipole moment, polarity, and kinetic diameter—becomes negligible. In this high-frequency regime, the capacitance is dominated by the rapid intrinsic dielectric polarization of HKUST-1 and the device’s geometric capacitance, making the response insensitive to VOCs whose slow kinetics cannot follow the oscillating field. Conversely, at lower frequencies, the capacitance becomes VOC-dependent and exhibits hysteresis, driven by slow interfacial and transport processes that occur on longer time scales.

Notably, the nonoverlapping error bars observed at lower frequencies demonstrate that AC response measurements provide a highly sensitive means of discriminating among different VOC atmospheres.

Our findings can be further understood by examining the diffusion dynamics of HKUST-1’s hierarchical pore structure (Figure 4b). While all tested molecules possess kinetic diameters well below the framework’s pore aperture (0.5–1.4 nm) — ensuring intrinsically favorable diffusion — the observed variation in hysteresis and capacitance responses is predominantly dictated by molecular polarity and dipolar interactions within the confined pore environment, rather than by size-related constraints alone.

Surface potential measurements further support this polarity-dependent behavior. The surface charge of the HKUST-1 thin-film was further assessed by KPFM (Figure S5), which revealed a uniform surface potential with a slight positive offset (∼80–100 mV) relative to the Au substrate. This positively polarized surface arises from the exposed Cu^2^^+^ paddlewheel sites and partially deprotonated carboxylate linkers, and it strongly influences molecular interactions with adsorbed species. Polar VOCs, such as methanol and ethanol, couple efficiently to these Lewis-acidic sites through dipole–dipole and Cu–O interactions, enhancing surface permeability and accelerating diffusion within the pores. In contrast, nonpolar VOCs like hexane interact weakly, yielding lower permeability (α) and diffusivity (D) values. This correlation confirms that the intrinsic surface charge distribution of HKUST-1 governs its selective and polarity-dependent adsorption behavior.

In the mass transfer processes, two mechanisms predominate: surface barrier and intracrystalline diffusion.^34,68^ The surface-barrier mechanism depends on (i) pore accessibility, (ii) surface defects (e.g., misaligned and defective pores), and (iii) surface chemical interactions. In contrast, intracrystalline diffusion governs the transport of molecules through internal crystalline channels after surface penetration,^69,70^ with kinetics determined by molecular mobility within the pore networks, pore geometry and connectivity, and guest-molecule interaction with the pore walls. While the fundamental origin of these mechanisms remains debated, Gao et al. proposed a quantitative theoretical approach to describe their combined effects:

$$\frac{m_{t}}{m_{\infty }}=1-\overset{\infty }{\underset{n=1}{\sum }}\frac{2L^{2}\exp\left(\frac{-\beta_{n}^{2}Dt}{l^{2}}\right)}{\left(\beta_{n}^{2}+L^{2}+L\right)\beta_{n}^{2}};\beta_{n}\tan\beta_{n}=L$$ 3

In eq 3, *m*_*t*_/*m*_∞_ is the relative uptake loading of guest molecules, *t* is the capture time, *l* is the characteristic length of intracrystalline diffusion, and *D* is the intracrystalline diffusivity (transport). *L* = α*l*/*D* characterizes the competition between intracrystalline diffusion and surface-barrier effects, where α represents the surface permeability (which controls molecular flux across the interface).

Applying eq 3 to evaluate intracrystalline diffusion poses a fundamental challenge because it requires prior knowledge of surface-barrier effects. To decouple these competing mechanisms, we employ a short-time approximation (*t* → 0) and apply the Laplace transformation to eq 3,^51^ yielding the analytically solvable equation:

$${\frac{m_{t}}{m_{\infty }}|}^{t\to 0}=\sqrt{\frac{4Dt}{{\pi l}^{2}}}-\frac{1-\exp\left(L^{2}Dt/l^{2}t\right)\mathrm{erfc}\left(L\sqrt{\frac{Dt}{l^{2}}}\right)}{L}$$ 4

Figure 4c presents the experimental transient current data plotted against the square root of time, along with corresponding curve fits derived from eq 4. The fitting procedure employed the Differential Evolution Algorithm (see Experimental Section for details), with all extracted parameters summarized in Table 1.

**Table 1:** Fitted Transport Parameters from Gao’s Model (Eq 4) for Each VOC

VOC	$\frac{\alpha }{l}[s^{-1}]$	*D* [m^2^/s]	*L* [u.a.]	*l* [Å]	α [m/s]
methanol	(1.71 ± 0.77) × 10^–14^	(2.04 ± 0.91) × 10^–13^	(2.08 ± 0.93) × 10^–8^	(7.31 ± 3.27) × 10^–11^	(3.25 ± 1.45) × 10^–11^
ethanol	(4.83 ± 2.16) × 10^–15^	(2.29 ± 1.02) × 10^–14^	(1.10 ± 0.49) × 10^–8^	(8.32 ± 3.72) ×10^–11^	(2.58 ± 1.15) × 10^–12^
propanol	(3.61 ± 1.61) × 10^–15^	(1.63 ± 0.73) × 10^–14^	(6.06 ± 2.71) × 10^–8^	(8.94 ± 4.00) × 10^–11^	(1.38 ± 0.62) × 10^–11^
hexane	(3.25 ± 1.45) × 10^–16^	(3.14 ± 1.40) × 10^–15^	(3.28 ± 1.47) × 10^–9^	(6.32 ± 2.83) × 10^–11^	(1.47 ± 0.66) × 10^–13^

The results reveal that the transport parameters — the intracrystalline diffusivity (*D*), surface permeability (α), and the surface barrier (expressed as α/*l*) — are critically dependent on both the intrinsic properties of the organic compounds and their interactions with the material’s surface. Among the studied molecules, methanol demonstrates the highest values for *D*, α, and α/*l* in HKUST-1. This behavior arises from methanol’s strong interactions with surface defects and acidic sites. While these interactions initially create a higher surface barrier, they simultaneously facilitate an alternative penetration pathway, ultimately enhancing efficiency. This alternative penetration pathway is primarily associated with missing linkers, open metal sites, and distortions.

In contrast, hexane — with its zero-dipole moment and low polarizability — exhibits the lowest values of *D*, α, and α/*l*. The lack of strong surface interactions hinders its efficient penetration of the pore network. While ethanol and propanol display comparable *D* values, propanol shows α roughly an order of magnitude higher than ethanol. This disparity likely arises from differences in molecular size and surface affinity. The relative contributions of surface and intracrystalline transport can be assessed using Gao’s model, where the characteristic diffusion length *L* = *D*/α, reflects the interplay between surface permeability (α) and bulk diffusivity (*D*). When *L* ≪ *d* (film thickness), molecular uptake is limited by the surface barrier, whereas *L* ≫ *d* indicates that intracrystalline diffusion dominates. In the present case, the extracted *L* values (∼10^–3^ to ∼10^–2^ m) are larger than the film thickness (∼7 × 10^–8^ m), confirming that mass transfer in the HKUST-1 layer is primarily governed by intracrystalline diffusion rather than surface resistance. This conclusion agrees with the high α values and the fully reversible transient current profiles observed for all VOCs. Notably, intracrystalline transport is primarily dictated by pore topology. It remains relatively consistent across molecules, whereas surface permeability is more sensitive to interfacial interactions, including steric effects and the distribution of acidic sites. The uniformity in *D* values supports the hypothesis that intracrystalline diffusivity is intrinsically tied to pore structure and molecular properties. Consequently, the observed contrast between methanol (fast diffusion) and hexane (slow diffusion) can be directly attributed to their kinetic diameters, highlighting how larger molecules face greater diffusion restrictions.

## Conclusions

In this study, we systematically investigated the diffusion dynamics of VOCs, including methanol, ethanol, propanol, and hexane, integrated HKUST-1 SURMOF-based vertical heterojunction devices using electrical characterization techniques. Our real-time monitoring approach revealed distinct diffusion behaviors, governed by molecular properties such as polarity, dipole moment, and kinetic diameter. Analysis of *I*–*t* and *C*–*f* responses demonstrated that polar interactions and dipole moments critically influence diffusion kinetics through dipolar and interfacial polarization mechanisms. Specifically, methanol exhibited the fastest diffusion and highest permeability, owing to its strong polarity, small kinetic diameter, and reduced confinement into the HKUST-1’s pores. In contrast, hexane, despite its higher volatility, displayed markedly slower diffusion due to its nonpolar nature and larger molecular size.

By applying Gao’s theoretical model, we quantitatively decoupled intracrystalline diffusion and surface-barrier effects. Notably, methanol’s superior diffusivity persisted even in the presence of higher surface barriers, underscoring the dominance of its intrinsic mobility and efficient pore permeation. Conversely, larger, nonpolar molecules, such as hexane, showed reduced permeability and slower diffusion, clearly limited by weak interfacial interactions.

These results provide a nanoscale understanding of VOC diffusion, emphasizing the interplay between molecular properties, pore geometry, and surface chemistry. Such insights advance the rational design of HKUST-1 and related SURMOFs for applications in selective molecular sensing, gas separation, and environmental or biomedical monitoring.

## Supplementary Material



## Notes

Experimental details, including device fabrication, schematic of layer-by-layer HKUST-1 SURMOF growth, rolled-up process, custom-built atmosphere-controlled chamber, complementary AFM/KPFM analysis, electrical characterization under inert and humid conditions, reproducibility tests, and diffusion analysis with Graham’s Law fitting parameters (PDF)

## Abbreviations

MOF: metal–organic framework
SURMOF: surface-supported metal–organic frameworks
VOCs: volatile organic compounds
