# Supplementary material for: Diffusion Dynamics of Volatile Organic Compounds into Integrated Surface-Supported Metal–Organic Frameworks Heterojunctions
Source: ACS Appl Mater Interfaces. 2025 Dec 22;18(1):2928–38. doi: 10.1021/acsami.5c16835 (PMC12781058; doi:10.1021/acsami.5c16835)
Supplement: Supplementary file 1 [file am5c16835_si_001.pdf]

Supporting Information for

Diffusion Dynamics of Volatile Organic Compounds into Integrated Surface-Supported Metal-Organic Frameworks Heterojunctions

Thamiris Cescon dos Santos[†,ⱡ,£], *Wagner Wlysses Rodrigues de Araujo*[†], *Tatiana Parra Vello*[ⱡ], *Carlos Vinícius Santos Batista*[†,£], *Luiz Gustavo Simão Albano*[†], *and Carlos César Bof Bufon*[£,§,*]

[†] Brazilian Nanotechnology National Laboratory (LNNano), Brazilian Center for Research in Energy and Materials (CNPEM), Campinas, São Paulo 13083-970, Brazil

[ⱡ] Mackenzie Institute for Research in Graphene and Nanotechnologies (MackGraphe), Mackenzie Presbyterian Institute (IPM), 01302-907, São Paulo, Brazil

[£] Postgraduate Program in Materials Science and Technology (POSMAT), São Paulo State University (UNESP), 17033-360, Bauru, São Paulo, Brazil

[§] Institute of Geosciences and Exact Sciences, São Paulo State University (UNESP), Rio Claro, São Paulo 13506-900, Brazil

*Corresponding author: cesar.bof@unesp.br

Summary

Device fabrication 3
Schematic illustration of layer-by-layer HKUST-1 SURMOF growth 4
Rolled-up process in detail 5
Custom-built atmosphere-controlled chamber 6
Complementary AFM and KPFM analysis 7
Electrical characterization for inert and humidity conditions 8
Electrical response reproducibility 9
Graham's Law and fitting parameters 10

Device fabrication

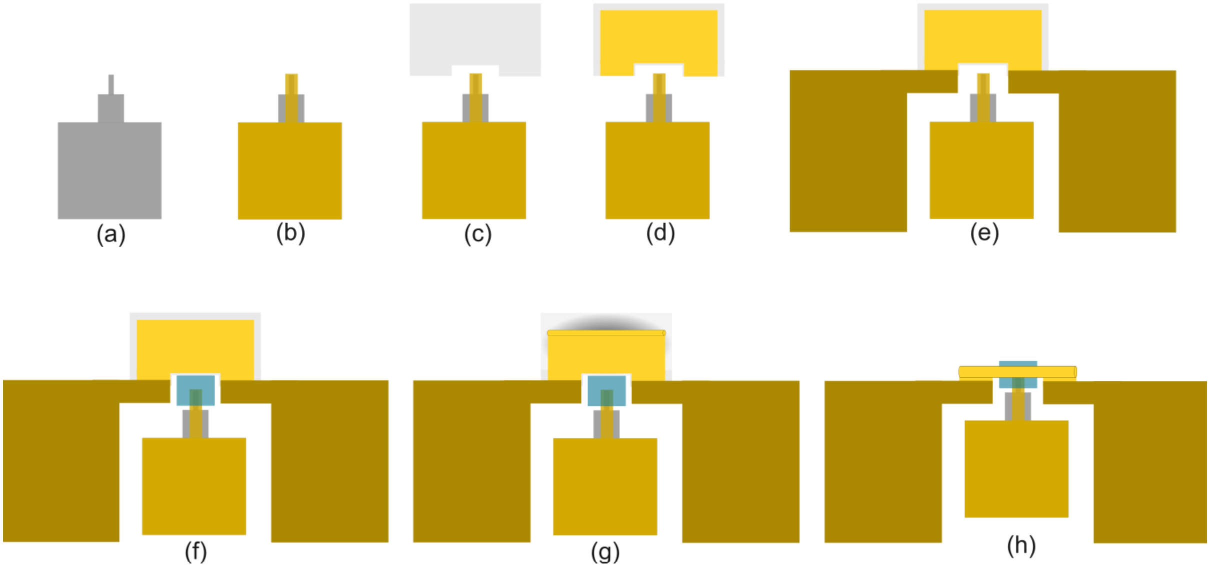

Figure S1. Schematic illustration from the main fabrication steps for vertical heterojunctions based on nanomembrane-origami technology. (a) Creation of SiO_2 mesa structure (190 nm height) using reactive ion etching process. (b) SiO_2 mesa structure covered with Cr/Au (5/10 nm) metallic layers, resulting in a finger-like (bottom) electrode. (c) Patterning and deposition of the Ge layer around the finger-like electrode, the subsequent oxidation process turns Ge into GeO_x, soluble into aqueous solution, and used as the sacrificial layer. (d) Patterning and deposition of strained metallic nanomembrane based on Au/Ti/Cr trilayer on GeOx. (e) Patterning and deposition of the contact pads based on Cr/Au (20/60 nm). (f) Patterning HKUST-1 SURMOF on the active device area after layer-by-layer growth. (g)-(h) Rolled-up process through selective GeO_x etching.

Schematic illustration of layer-by-layer HKUST-1 SURMOF growth

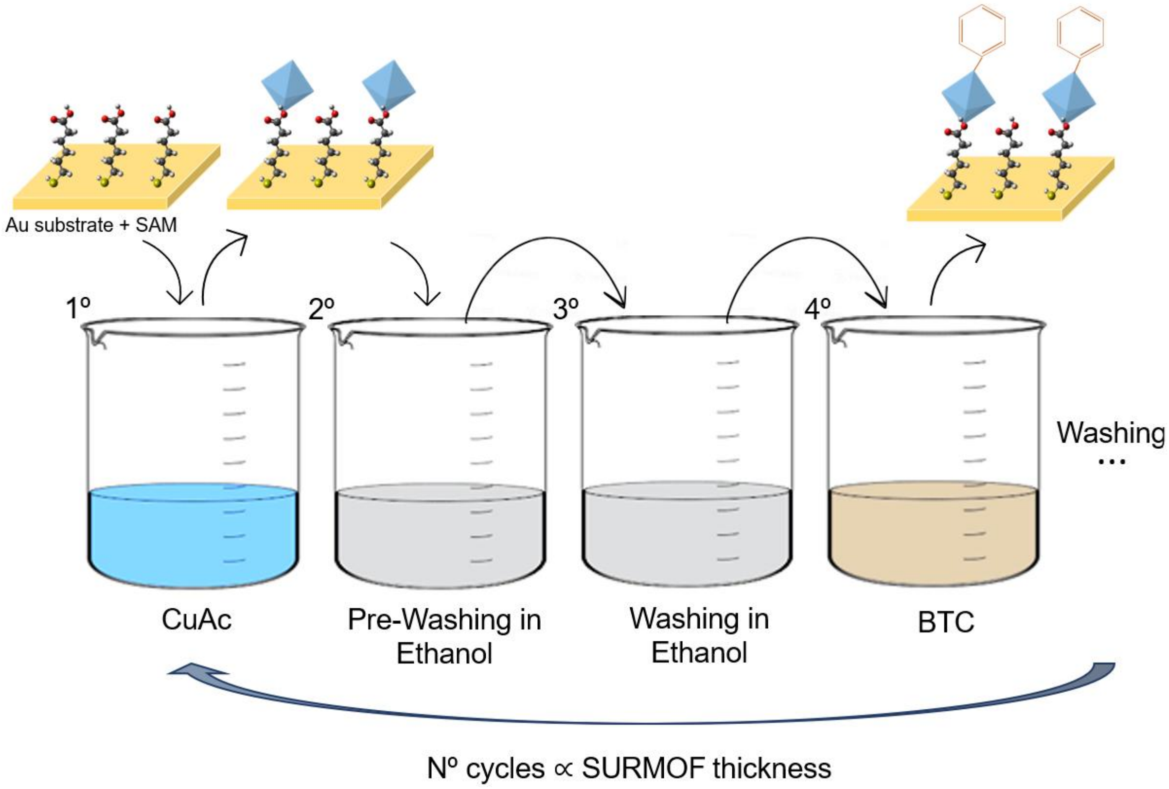

Figure S2. Schematic illustration of layer-by-layer HKUST-1 SURMOF growth based on a quasi-liquid-phase epitaxial process. The gold (Au) substrate functionalized with a self-assembled monolayer (SAM) is immersed into an ethanolic solution containing metallic clusters (CuAc, 1º), followed by the immersion into two sequential cleaning ethanolic solutions (2º and 3º), and then into ethanolic solution containing organic ligands (BTC, 4º), completing one cycle of deposition. The thickness can be precisely adjusted through the number of cycles.

Rolled-up process in detail

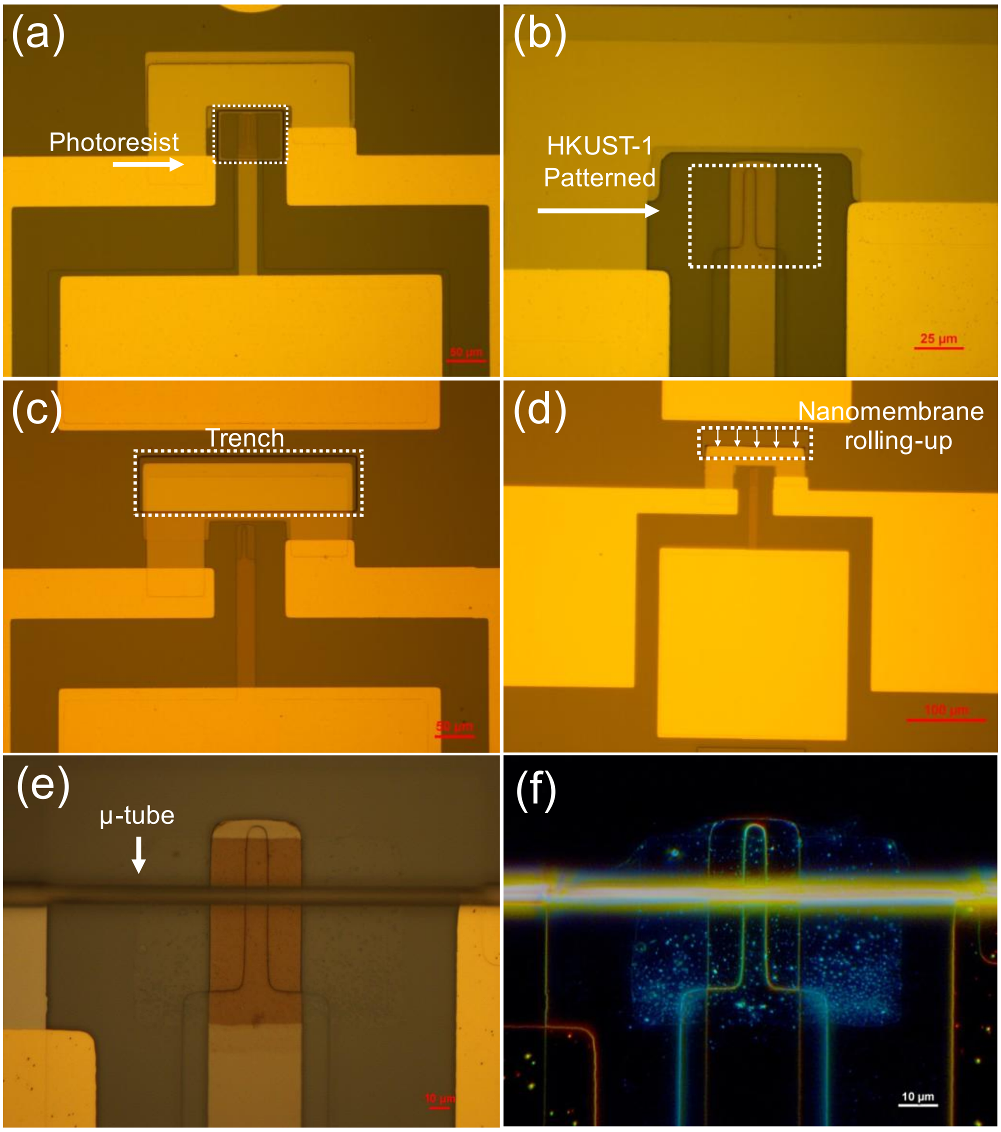

Figure S3. Additional optical microscope images from HKUST-1 SURMOF patterning and rolled-up process. (a) Photoresist μ-thick layer protecting HKUST-1 SURMOF from the etching process on the active device area. (b) Patterned HKUST-1 SURMOF after photoresist dissolution. (c) Patterning of a trench structure on a strained metallic nanomembrane inside the trench region remains without photoresist. (d) Selective dissolution of the GeO_x sacrificial layer guided by the trench structure, enabling the strained metallic nanomembrane to curl and roll reproductively. Dotted regions in (a), (b), (c), and (d) show the areas of interest. (e) After trench removal, the rolled-up process is completed, creating a μ-tube (top) electrode in soft contact with HKUST-1 SURMOF. (f) Dark-field image obtained with optical microscopy from (e).

Custom-built atmosphere-controlled chamber

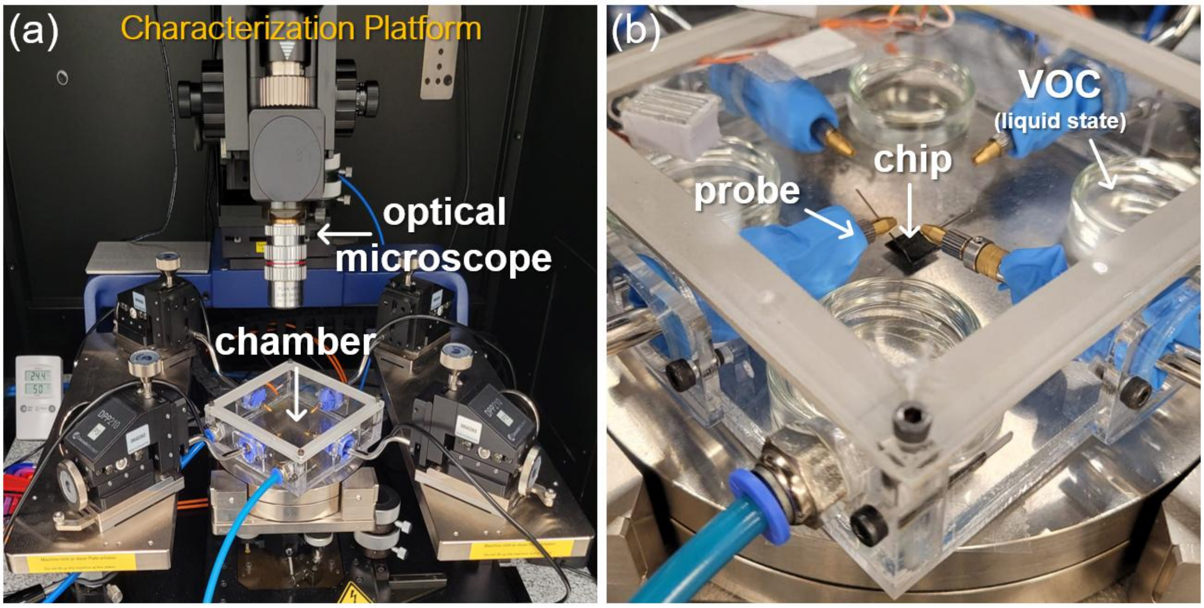

Figure S4. Photographs from customized electrical measurement system. (a) Custom-built atmosphere-controlled chamber with N_2 flow integrated into the conventional probe station setup. (b) A detailed photograph of the chamber shows the four liquid reservoirs for different VOCs. For humidity control, a digital monitor reads the nominal values, which can be regulated through N_2 flow regulation.

Complementary AFM and KPFM analysis

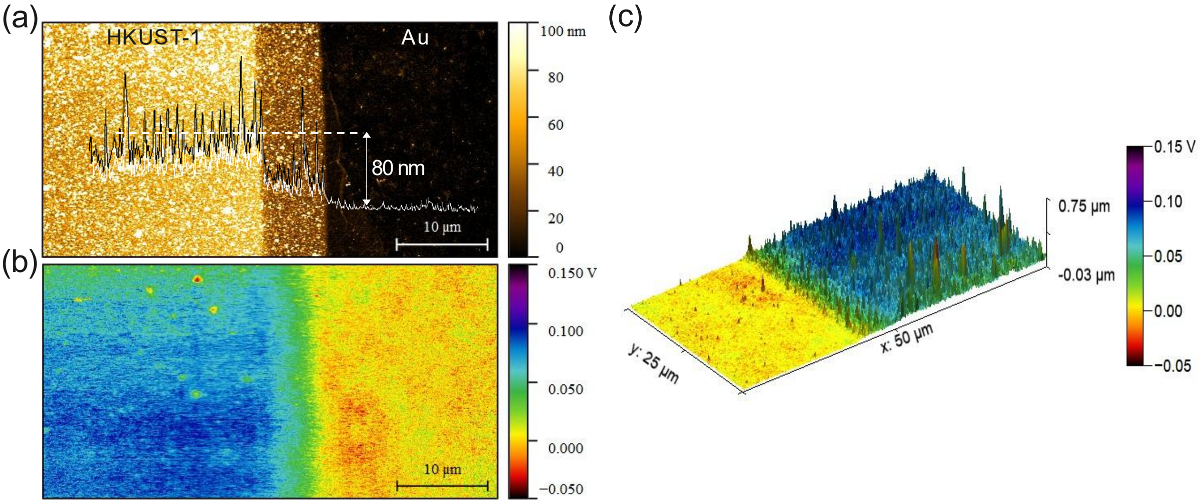

Figure S5. Additional AFM and KPFM results for HKUST-1 SURMOF. (a) AFM topography image and profile height for HKUST-1 SURMOF deposited on Au. (b) Respective KPFM surface potential map, showing distinct surface electrical profiles. (c) Three-dimensional representation of (b).

Electrical characterization for inert and humidity conditions

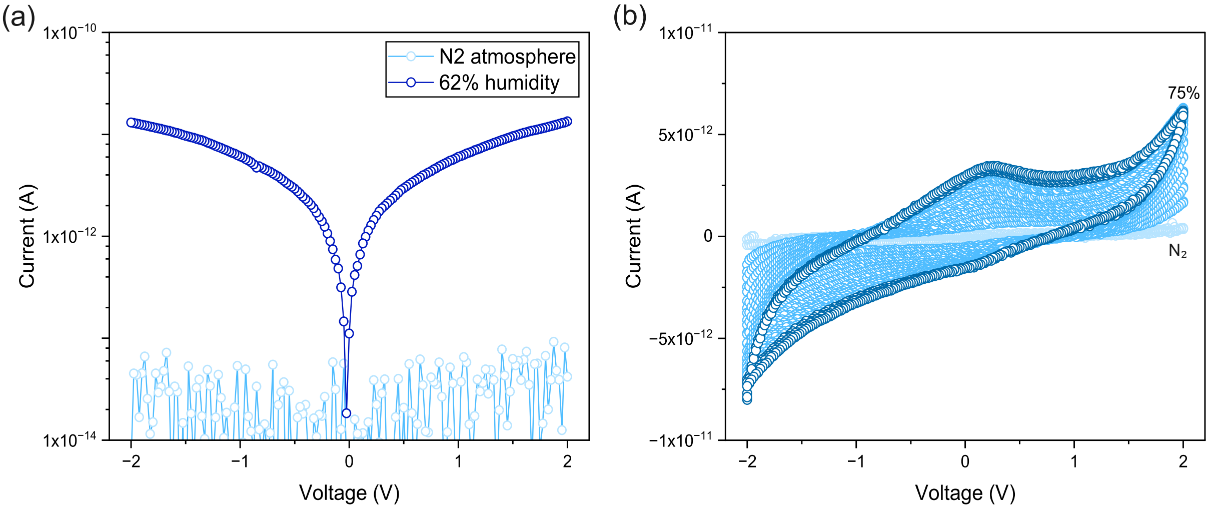

Figure S6. Typical electrical current vs applied voltage characteristics for inert and humidity conditions. (a) Comparative curves for inert and 62 % of relative humidity conditions. In the first condition, the HKUST-1 behaves as an intrinsically insulating material, whereas under moderate relative humidity, the electrical current increases up two orders of magnitude. (b) Series of curves from the same device, starting for the inert atmosphere and gradually increasing the relative humidity until reaching approximately 75 %.

Electrical response reproducibility

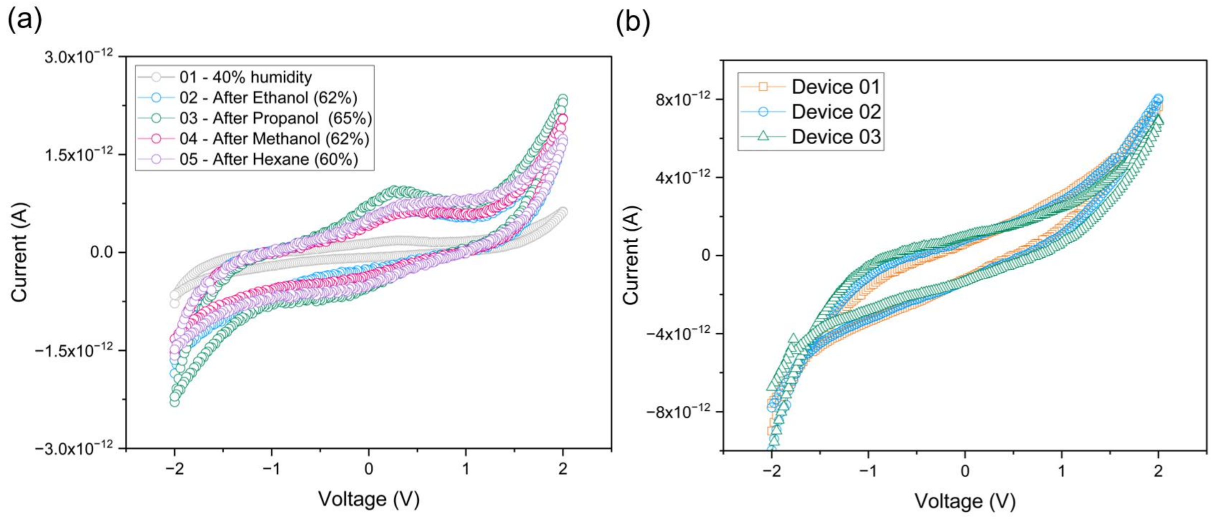

Figure S7. Typical electrical current vs applied voltage characteristics for pristine devices. (a) Curves from different devices before VOC loading. (b) Standard electrical response at humidity of 70 % for a triplicate set of devices.

Graham's Law and fitting parameters

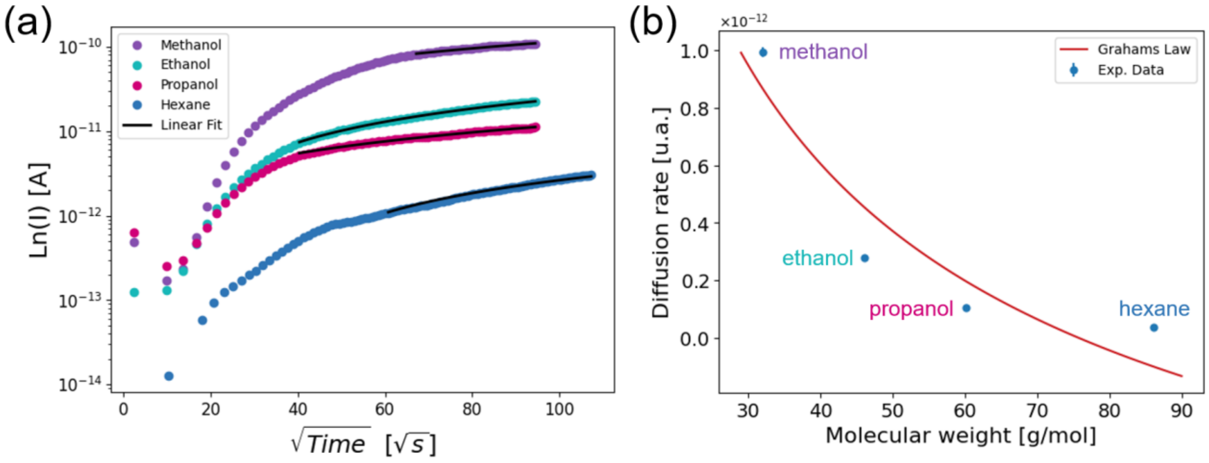

Figure S8. Typical curves to obtain diffusion parameters. (a) Natural logarithm of electrical current versus square root of time obtained from transient curves (I x t, Figure 3e) for different VOCs. (b) Diffusion rate versus molecular weight for different VOCs (solid circles) and Graham's Law prediction (line).

Table S1. Fitting parameters obtained from the linear data.

VOC	Slope (u.a.)	Error Y (u.a.)	Linear Fit (R^2)	Graham's (R^2)
Methanol	$9.94x10^{-13}$	$1.65x10^{-14}$	0.99	0.87
Hexane	$3.90x10^{-14}$	$4.45x10^{-16}$	0.99	0.87
Ethanol	$2.80x10^{-13}$	$7.94x10^{-16}$	0.99	0.87
Propanol	$1.10x10^{-13}$	$8.44x10^{-16}$	0.99	0.87
